# Level Set Based Hippocampus Segmentation in MR Images with Improved Initialization Using Region Growing

**DOI:** 10.1155/2017/5256346

**Published:** 2017-01-15

**Authors:** Xiaoliang Jiang, Zhaozhong Zhou, Xiaokang Ding, Xiaolei Deng, Ling Zou, Bailin Li

**Affiliations:** ^1^College of Mechanical Engineering, Quzhou University, Quzhou, Zhejiang 324000, China; ^2^College of Mechanical Engineering, Southwest Jiaotong University, Chengdu, Sichuan 610031, China; ^3^Department of Radiology, West China Hospital, Sichuan University, Chengdu, Sichuan 610031, China

## Abstract

The hippocampus has been known as one of the most important structures referred to as Alzheimer's disease and other neurological disorders. However, segmentation of the hippocampus from MR images is still a challenging task due to its small size, complex shape, low contrast, and discontinuous boundaries. For the accurate and efficient detection of the hippocampus, a new image segmentation method based on adaptive region growing and level set algorithm is proposed. Firstly, adaptive region growing and morphological operations are performed in the target regions and its output is used for the initial contour of level set evolution method. Then, an improved edge-based level set method utilizing global Gaussian distributions with different means and variances is developed to implement the accurate segmentation. Finally, gradient descent method is adopted to get the minimization of the energy equation. As proved by experiment results, the proposed method can ideally extract the contours of the hippocampus that are very close to manual segmentation drawn by specialists.

## 1. Introduction

**Figure 1:**
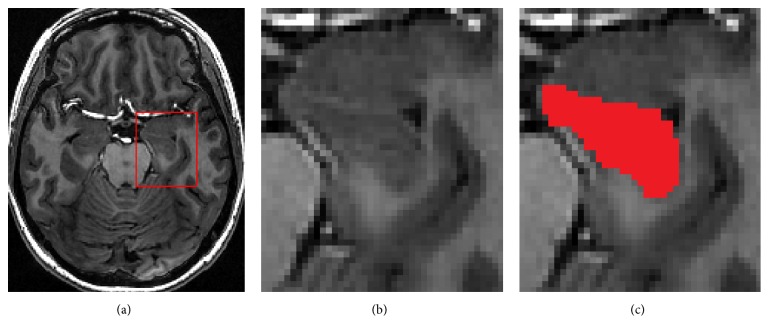
Position and intensity of hippocampus in MR image. (a) Original image of hippocampus with a rectangle; (b) zoomed view of the rectangle region; (c) zoomed view of the region where the hippocampus is indicated with red color.

Magnetic Resonance Imaging (MRI) is a noninvasive medical imaging technology which provides anatomical information on disease-related for detection of pathology and treatment planning. Many clinical applications depend on the segmentation results of MRI brain structures that allow us to know our brain anatomy changes during the deterioration of disease. However, accurate and reliable automatic segmentation of medial temporal lobe structures, such as the hippocampus, is still a challenging. Hippocampus, being one of a small temporal lobe brain structure, has been found to be highly related to many neurological pathologies and psychiatric disorders including Alzheimer's disease [1, 2], epilepsy [3, 4], schizophrenia [5, 6], and mild cognitive impairment [7]. Unfortunately, at the spatial resolution of MRI, the structure of hippocampus has relatively low contrast and no distinguishable boundaries, as shown in Figure 1. Traditionally, clinical hippocampus segmentation almost relies on the manual, which is rater-dependent and highly time-consuming. So, how to segment hippocampus automatically in medical image analysis is critical in clinical aspects.

In recent years, many scholars have been engaged in hippocampus segmentation algorithm, and various types of works also have been done. For example, Kim et al. [8] built a surface model of hippocampus and used principal component analysis to find the shape asymmetry of hippocampus between schizophrenic patients and healthy controls. Wang et al. [9] proposed a region growth algorithm based on seed which is simple and effective but could fail due to the indistinct boundary of hippocampus. Tong et al. [10] proposed a new method for nearly automated detection of the hippocampus contours on MRI images based on combined sparse coding techniques and discriminative dictionary learning. Hajiesmaeili et al. [11] utilized contraction wave atom as an efficient method for enhancing the noisy of MR images to improve segmentation accuracy. Kwak et al. [12] presented graph cuts algorithm to segment hippocampus. Similarly, Wolz et al. [13] applied a graph cuts algorithm to simultaneously measure hippocampal atrophy in longitudinal studies.

Another hotspot of segmentation approach is level set method. It allows integration of prior information within a single optimization framework and has proved to be powerful tools in image segmentation. One popular example of edge-based methods is the Geometric Active Contour (GAC) model [14], which used the image gradient to stop evolving contours on object boundaries. The Chan-Vese model [15], which based on the Mumford-Shah segmentation framework [16], is one of the most popular region-based models to detect objects whose boundaries are not defined by strong edges. Recently, Li et al. [17] proposed a new edge-based variational level set method without reinitialization. It incorporated a penalty term to preserve the level set function close to a signed distance function. However, these previous works usually present some problems such as oversegmentation and lower speed.

In this paper, we propose a level set framework combined with seeded region growing algorithm for the segmentation of hippocampus. The method has several advantages: (1) the outcome of the region growing approach is provided automatically as the initial contour of level set evolution method; (2) the global Gaussian distribution with different means and variances is integrated into level set framework. By effectively combining region growing and level set algorithm, the segmentation accuracy is improved while the processing time is decreased. The remaining part of the paper is organized as follows. In Section 2, the proposed method and its theoretical background is presented. In Section 3, results and discussion are given. In Section 4, we state our conclusion.

## 2. Methods

**Figure 2:**
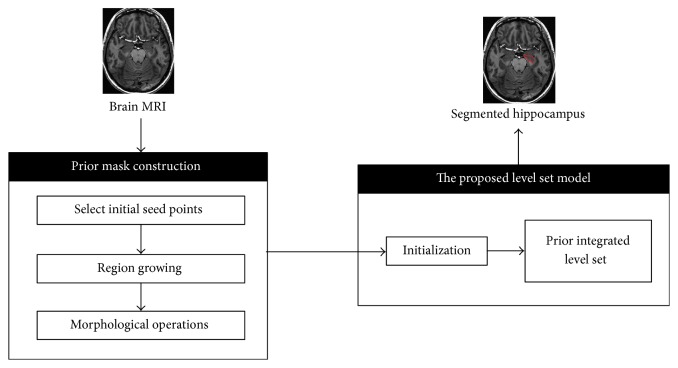
Block diagram of the proposed hippocampus segmentation approach.

Level set method is defined as energy minimization process which can be applied for image segmentation. However, traditional level set algorithm has some commonly known weaknesses: (1) the initial contour is difficult to extract; (2) for high intensity inhomogeneity and discontinuous boundaries images, the edge stop function will produce high values and the contour may be attracted to false edges. In this section, an accurate and efficient level set algorithm is presented. Firstly, the evolution of the level set model is initialized using region growing as the prior information. Besides that, we integrated the global Gaussian distributions information into a conventional edge-based level set model, proposed in [17]. The role of the edge-based is to guide segmentation towards apparent boundaries, while the global Gaussian distributions term is to take lead on regions with weak boundaries. The detail of the proposed segmentation approach is described in Sections 2.1to 2.2. The flowchart of the proposed method is shown in Figure 2.

### 2.1. Region Growing

Traditional level set algorithms can handle the segmentation problem accurately by using some contour or image dynamics such as curvature, intensities, or derivatives. But all approaches need the initialization step so that the automatic operation of the system is difficult to achieve. Consequently, some researchers have used shape-based methods to segment the hippocampus and proposed local solutions strictly depending on the shape information of the interest. In this section, the outcome of the region growing approach is provided automatically as the initial contour to the distance regularized level set method. The contour based Li's method [17] is thus aided by region growing approach and improves the segmentation performance compared to both the region growing and Li's models.

The region growing [18–20] is a simple region-based image segmentation method that attempts to segment images into many small regions based on predefined seed points, grow rule, and stop condition. As the name is implied, region growing starts from the seed points to the adjacent pixels according to the similarity between the pixel and the marked region and then determines whether the pixel neighbors should be added to the regions. Obviously, selecting seed points and the growth rules is the key of regional growth. However, this method may encounter difficulties with images in which regions both are noisy and have blurred or indistinct boundaries. Therefore, in this study, we proposed a novel and adaptive region growing method for semiautomatically generating the preliminary boundary of hippocampus in MRI. Detailed process will be introduced as follows.

**Step 1 .**

**Figure 3:**
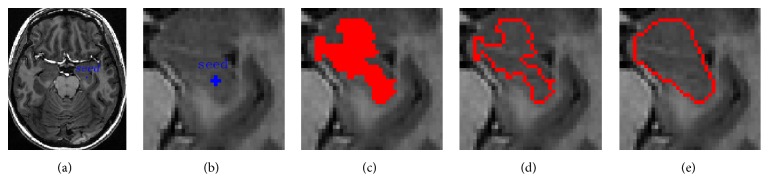
Initialization procedure of the level set function using region growing. (a) Original images of hippocampus with initial seed points (blue); (b) slice images of hippocampus; (c) homogeneous region as a result of region growing (red); (d) perimeter of the homogeneous region (red); (e) convex polygon fitting of the perimeter, used as zero level set (red).

**Figure 4:**
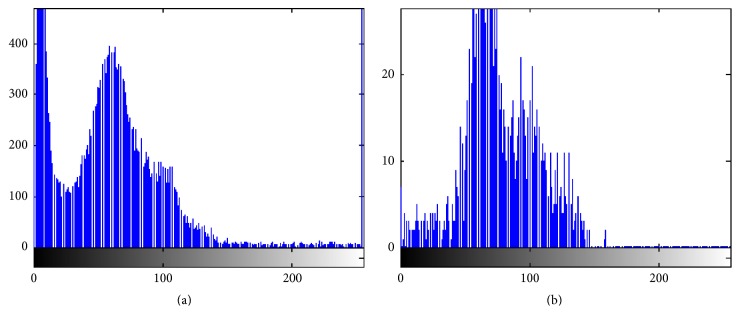
Comparison of gray histogram between original image and slice image. (a) The gray-level histogram of original image; (b) the gray-level histogram of slice image.

Select initial seed points: in MRI images, the hippocampus structure has similar gray values with its adjacent anatomical structures and it is only a small region. In order to reduce the computation and improve the segment reliability, we only process a local area, including the whole hippocampus and little other region. Firstly, an initial seed point is manually selected on the original image of hippocampus, as shown in Figure 3(a). Then, we obtain slice image, with the size of 45*∗*45 pixels, of which center is the initial seed point position, as shown in Figure 3(b). After shearing, the majority of the cerebrospinal fluid, white matter, and gray matter can be filtered out, so that it can reduce the interference of the hippocampus segmentation. From the gray histogram shown in Figure 4, we can see that the slice images can better reflect the characteristics of the hippocampus and improve the segmentation accuracy.

**Step 2 .**

Region Growing: given the seed, regions are grown from it by appending those neighboring pixels whose properties are most similar to the region. According to the special position of hippocampus in MR image, we choose the standard deviation as the growth rule. It can be described as follows:

(1) $$\begin{array}{c} \left|\;I\left(\;x,y\;\right)-{\overset{-}{X}}_{\text{seed}}\;\right|\leq \text{threshold}=\xi \sigma_{\text{Global}}, \end{array}$$

where *I*(*x*, *y*) is the slice image, ${\overset{-}{X}}_{seed}$ is the mean intensity of current seed points, *σ*_Global_ is the global standard deviation of the slice image, and *ξ* is the weight of *σ*_Global_. This criterion allows including all connected pixel blocks, obtained by the quad-tree decomposition, of which intensity does not differ from the seed point intensity more than a user-defined threshold value. Based on this observation, *σ*_Global_ can be defined as

(2) σGlobal=1M×N∑x=1M∑y=1NIx,y−X−2,X−=1M×N∑x=1M∑y=1NIx,y,

where *M* × *N* is the size of slice image and $\overset{-}{X}$ is the mean intensity of slice image. If any pixel belonging to the seed point's 8-connected neighborhood satisfies the growth criterion, we add it to the seed point set. The experiment results of our region growing algorithm are presented in Figure 3(c).

**Step 3 .**

Morphological operations: morphological operations are a collection of nonlinear operations related to the shape or morphology of features in an image. Firstly, the perimeter of the homogeneous region obtaining by region growing is extracted, as shown in Figure 3(d). However, the result is not the hippocampus region we desire to extract exactly. Therefore, level set method is introduced to modify the result which is produced by region growing. In order to prevent falling into local minima, a convex polygon is gained by fitting the perimeter and used to initialize the level set algorithm, as shown in Figure 3(e).

### 2.2. Level Set Algorithm

#### 2.2.1. Traditional Level Set Algorithm

Level set methods have been widely used for image segmentation [21–26]. In general, these methods can be broadly classified as either edge-based or region-based. In implementing the traditional level set methods, it is numerically essential to keep the evolving level set function close to a signed distance function. This process is called reinitialization, and it can be quite complicated and time-consuming. In [17], Li et al. proposed a new variational formulation of level set method without reinitialization. The energy functional is defined as

(3) εϕμPϕ+λLgϕ+νAgϕ=12μ∫Ω∇ϕ−12dx dy+λ∫Ωgδεϕ∇ϕdx dy+ν∫ΩgHε−ϕdx dy,

where *μ*, *λ* > 0, *ν* are vary constants and *ϕ* is a level set function. The *P*(*ϕ*) is the penalizing term, *L*_*g*_(*ϕ*) is the length of the interface, *A*_*g*_(*ϕ*) is the area of the subregion enclosed by the zero level curve, the function *H*_*ε*_(·) and *δ*_*ε*_(·) are the Heaviside and Dirac function, and *g* is an edge indicator function, defined by

(4) $$\begin{array}{c} g=\frac{1}{1+{\left|\nabla G_{\sigma }*I\right|}^{2}}, \end{array}$$

where *G*_*σ*_ is the Gaussian kernel having the standard deviation *σ*.

Due to the penalizing term *P*(*ϕ*), the involving level set function driven by (3) can automatically close to a signed distance function; thus reinitialization procedure is eliminated completely. However, this method above has at least three intrinsic limitations in applications. First, it is highly sensitive to the contour initializations; therefore we have to choose suitable initial contours to correctly detect object of interest in given images. Second, it is prone to getting “trapped” by extraneous edges caused by image noise or concavity caused by high intensity variations near these points. Third, it only can detect objects with edges defined by gradient.

#### 2.2.2. The Proposed Level Set Algorithm

As discussed in the above section, Li's method is highly sensitive to strong noise and cannot extract edge without gradient or cognitive edges. For the accurate and efficient detection of the hippocampus, we use the region growing as the prior information and integrate the global Gaussian distributions information into a conventional edge-based level set model. In image segmentation, the role of global information plays a leading on regions with weak boundaries. Particularly it is crucial to reduce the sensitivity to the initialization of the contour. From statistical point of view, in the proposed model, the fitting energy is described by a combination of Li's model and global Gaussian distributions with different means and variances, respectively. The global Gaussian distributions fitting energy is defined as follows:

(5) $$\begin{array}{c} E^{GDF}=\overset{2}{\underset{i=1}{\sum }}\int_{\Omega_{i}}-\log p_{i}\left(\;I\left(\;x\;\right)\;\right)dx,\;i=1,2, \end{array}$$

where *Ω*_1_ = in(*C*), *Ω*_2_ = out(*C*), and *p*_*i*_(*I*(*x*)) is

(6) $$\begin{array}{c} p_{i}\left(\;I\left(\;x\;\right)\;\right)=\frac{1}{\sqrt{2\pi }\sigma_{i}}\exp\left(\;-\frac{{\left(I\left(x\right)-u_{i}\right)}^{2}}{2\sigma_{i}^{2}}\;\right), \end{array}$$

where *u*_*i*_ and *σ*_*i*_ are global intensity means and standard deviation, respectively.

Let *Ω* ⊂ *R*^2^ be a two-dimensional image space and let *I* : *Ω* → *R* be a given gray image. We assume that the image domain can be partitioned into two regions. These two regions can be represented as the regions outside and inside the zero level *ϕ*; that is *Ω*_1_ = {*ϕ* > 0} and *Ω*_2_ = {*ϕ* < 0}. Using the Heavside function *H*_*ε*_, the global Gaussian distribution fitting energy function can be rewritten as follows:

(7) EGDFϕx=∫Ω−log⁡p1IxHεϕxdx+∫Ω−log⁡p2Ix1−Hεϕxdx.

In this study, we define the energy function as follows:

(8) Fϕx=μPϕx+λLgϕx+νAgϕx+τEGDFϕx=12μ∫Ω∇ϕx−12dx+λ∫Ωgδεϕx∇ϕxdx+ν∫ΩgHε−ϕxdx+τ∫Ω−log⁡p1IxHεϕxdx+∫Ω−log⁡p2Ix1−Hεϕxdx,

where *τ* is weighting constants of *E*^GDF^.

By calculus of variations, it can be shown that the parameters *u*_*i*_ and *σ*_*i*_^2^ that minimize the energy functional in (8) satisfy the following Euler-Lagrange equations:

(9) ∫Ix−uiMi,εϕxdx=0,∫σi2−Ix−ui2Mi,εϕxdx=0,

where *M*_1,*ε*_(*ϕ*(*x*)) = *H*_*ε*_(*ϕ*(*x*)) and *M*_2,*ε*_(*ϕ*(*x*)) = 1 − *H*_*ε*_(*ϕ*(*x*)).

From (9), we obtain

(10) ui=∫IxMi,εϕxdx∫Mi,εϕxdx,

(11) σi2=∫Ix−ui2Mi,εϕxdx∫Mi,εϕxdx,

which minimize the energy functional *F*(*ϕ*) for fixed *ϕ*.

Minimization of the energy functional (8) with respect to *ϕ* can be achieved by solving the gradient descent flow equation:

(12) ∂ϕ∂t=μΔϕ−div⁡ΔϕΔϕ+λδεϕdiv⁡gΔϕΔϕ+νgδεϕ−τδεϕe1−e2,

where

(13) e1=log⁡2πσ1+Ix−u122σ12,

(14) e2=log⁡2πσ2+Ix−u222σ22,

(15) Hεx=121+2πarctan⁡xε,

(16) δεx=1πεε2+x2.

#### 2.2.3. Algorithms and Numerical Approximation

In this subsection, we briefly present the numerical algorithms and procedures to solve the evolution (12). Due to the regularization term *P*(*ϕ*), (12) in the continuous domain can be discretized by means of simple finite difference rather than complex upwind difference scheme. We recall first the usual notation. Let *h* be the space step, let Δ*t* be the time step, and let (*x*_*i*_, *y*_*i*_) = (*ih*, *jh*) be the grid points. Let *ϕ*_*i*,*j*_^*n*^ = *ϕ*(*n*Δ*t*, *x*_*i*_, *y*_*j*_) be an approximation of the level set function *ϕ*(*t*, *x*, *y*), with *n* ≥ 0, *ϕ*^0^ = *ϕ*_0_, where *ϕ*_0_ is the initial level set function. The central differences of spatial partial derivatives are expressed in the following notations:

(17) Δ0xϕi,j=ϕi+1,j−ϕi−1,j2h,Δ0yϕi,j=ϕi,j+1−ϕi,j−12h.

All the spatial partial derivatives ∂*ϕ*/∂*x* and ∂*ϕ*/∂*y* are approximated by the central difference, and the temporal partial derivative ∂*ϕ*/∂*t* is discretized as the forward difference. Set *n* = 0 and start with initial level set function *ϕ*_*i*,*j*_^0^; knowing *ϕ*_*i*,*j*_^*n*^, we first compute *e*_1_^*n*^ and *e*_2_^*n*^ according to (13) and (14). Then, the numerical approximation to (12) is given by the following discretization:

(18) ϕi,jn+1−ϕi,jnΔt=μΔϕi,jn−ki,jn+λδεϕi,jn·gki,jn+Δ0xgΔ0xϕi,jn∇ϕi,jn+Δ0ygΔ0yϕi,jn∇ϕi,jn+νgδεϕi,jn−τδεϕi,jne1n−e2n,

where the corresponding curvature *k*_*i*,*j*_^*n*^ can be discretized using a second-order central difference scheme:

(19) $$\begin{array}{c} k_{i,j}^{n}=\Delta_{0}^{x}\left(\;\frac{\Delta_{0}^{x}\phi_{i,j}^{n}}{{\left|\nabla \phi \right|}_{i,j}^{n}}\;\right)+\Delta_{0}^{y}\left(\;\frac{\Delta_{0}^{y}\phi_{i,j}^{n}}{{\left|\nabla \phi \right|}_{i,j}^{n}}\;\right), \end{array}$$

with

(20) $$\begin{array}{c} {\left|\;\nabla \phi \;\right|}_{i,j}^{n}=\sqrt{{\left(\Delta_{0}^{x}\phi_{i,j}^{n}\right)}^{2}+{\left(\Delta_{0}^{y}\phi_{i,j}^{n}\right)}^{2}}. \end{array}$$

Next, a direct implementation of the proposed level set algorithm is presented in Algorithm 1.

## 3. Results and Discussion

To assess the performance of the proposed method, quantitative and visual experiments have been carried out on three groups of the whole brain MRI images (the image size is 157*∗*189 and 2D axial brain images with T1-weighted sequence of size 0.78*∗*0.78*∗*2.00 mm). For each image set, 17 to 19 frames which correctly resemble the shape of hippocampus were used to make the model. These images are from West China Hospital, Sichuan University, China. Li's model and the method of this paper are applied to the hippocampus brain segmentation, while the experiment results were compared. Two methods set the same parameters and initial conditions as follows: the level set function *ϕ* can be simply initialized as a binary value −*c*_0_ inside the region and a positive value *c*_0_ outside the region. Unless otherwise specified, we use the following default setting of the parameters in the experiments: time step Δ*t* = 4, *c*_0_ = 2, *μ* = 0.05, *λ* = 10, *ν* = 2, *τ* = 0.01, *ε* = 2, and *σ* = 1. The results are all carried out by Matlab2011a on the PC with Pentium CPU 2.50 and 4 GB of RAM, Windows 7 64 bits.

### 3.1. Segmentation of Hippocampus Images

**Figure 5:**
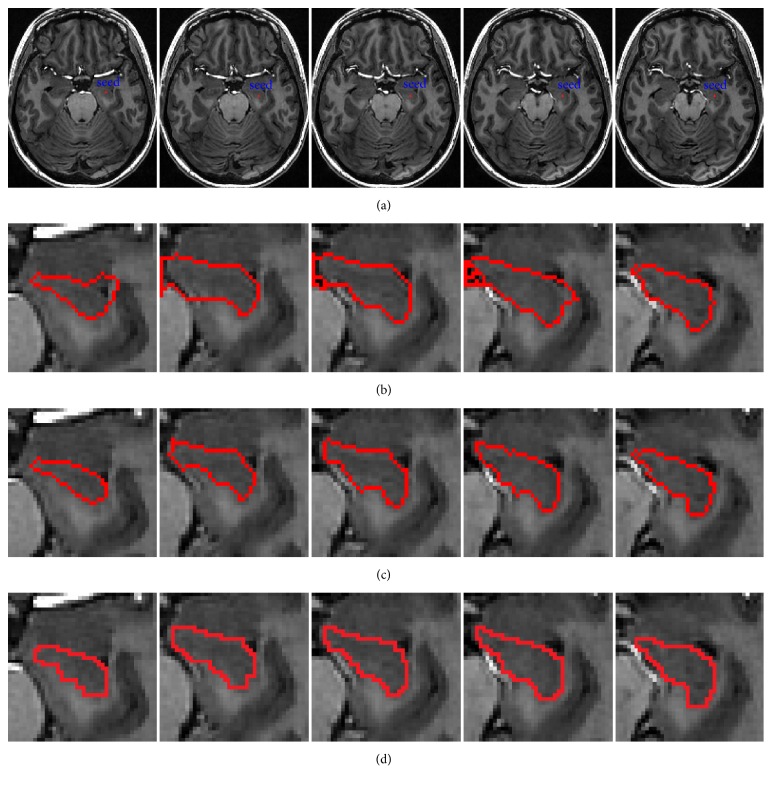
Segmentation of hippocampus with different algorithms. (a) Five original images of hippocampus with initial seed points (blue). (b) Results of Li's model (red). (c) Results of our method (red). (d) Results of artificial segmentation by the specialist (red).

Figure 5 offers the segmentation results of five hippocampus images, where the red curve surrounded is the segmentation area. Our method requires a seed point located inside the targeted structure by the user, as shown in Figure 5(a). The segmentation results by Li's model and our method are shown in the second row and the third row, respectively. The last row is the results of artificial segmentation by specialist. The experiment results with the same seed points show that when the images are badly under the conditions of intensity inhomogeneity, low contrast, and discontinuous boundaries, only using the edge information may fail to discriminate the intensity between an object and background, leading to inaccurate segmentation. However, our method both uses the edge information and region information of images, making our method have a very strong discriminative capability for the object and background. So, the segmentation results of this paper are more accurate, which can distinguish regions similar intensity means but different variances.

**Figure 6:**
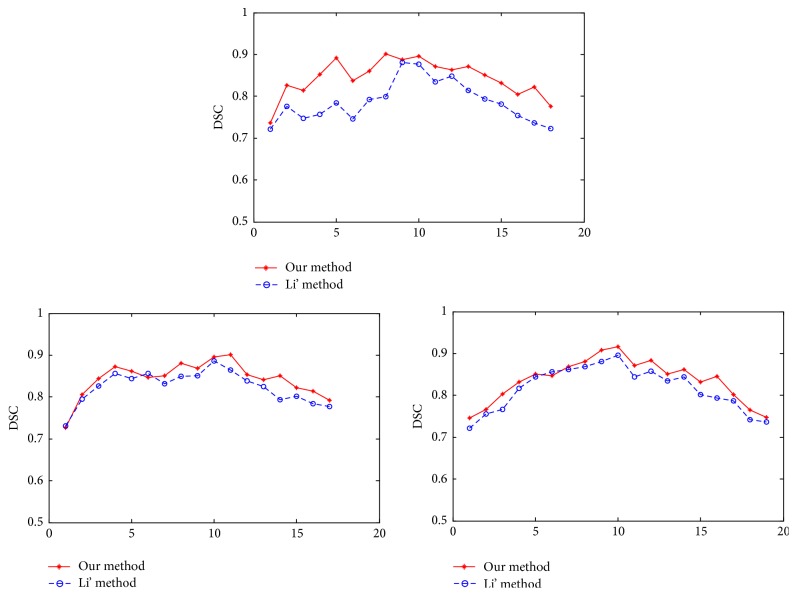
Quantitative comparison of our method with Li's model using the DSC standards from three different group MRI images.

Furthermore, in order to show the superiority of our model, we utilize the dice similarity coefficient (DSC) [27] to evaluate the performances on three different group images. If *S*_1_ and *S*_2_ stand for the areas enclosed by contours obtained by the model and the manual method respective, then the metric is defined as follows:

(21) $$\begin{array}{c} DSC=\frac{2N\left(S_{1}\cap S_{2}\right)}{N\left(S_{1}\right)+N\left(S_{2}\right)}, \end{array}$$

where *N*(·) indicates the number of pixels in the enclosed region and *Ω* is the image domain. The value of DSC ranges from 0 to 1, with a higher value representing a more accurate segmentation result. A comparison plot of DSC for the three groups of the whole brain MRI images which correctly resemble the shape of hippocampus is provided in Figure 6. By quantitative comparison, we can see from the overlap ratios that there is a very good correspondence between our method and manually extracted boundaries and this demonstrates the significant advantage of our model in terms of segmentation accuracy.

**Figure 7:**
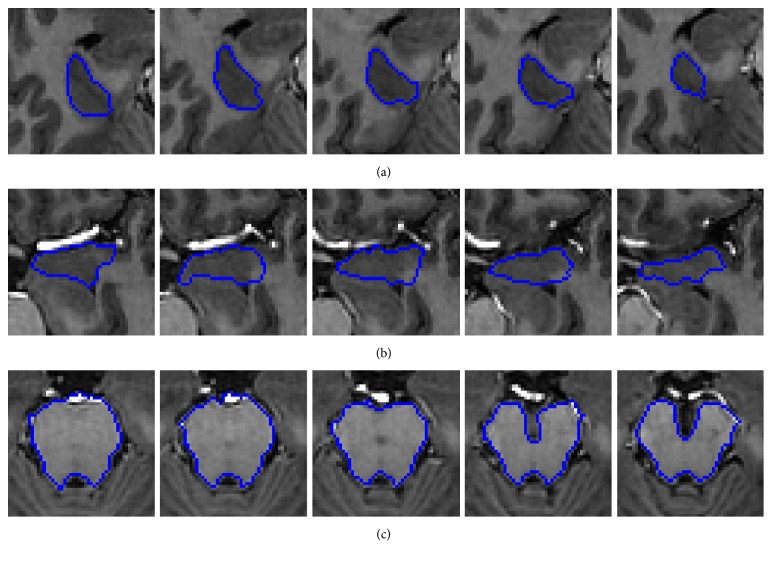
Segmentation results of our method on other brain tissue images.

In addition, Figure 7 shows the results of our method on other brain tissue images. The first to last rows show temporal lobe, amygdale, and midbrain, respectively. It can be observed that the good result can be obtained by our method.

### 3.2. Robustness to the Initial Seed Point

**Figure 8:**
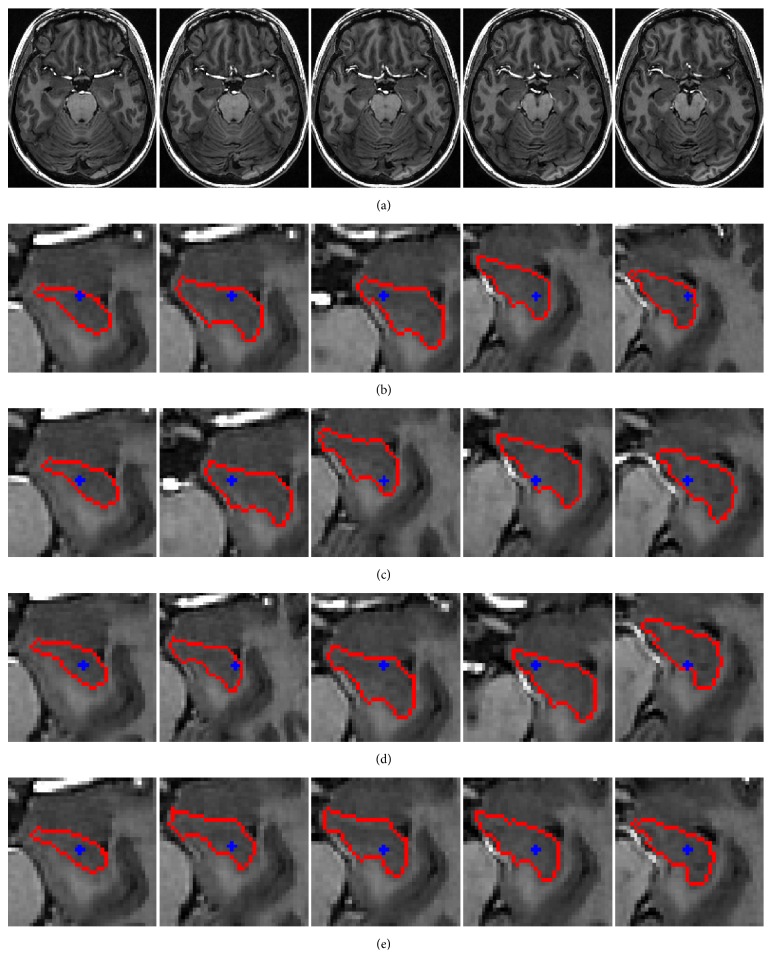
Segmentation of hippocampus with different initial seed points. (a) Five original images of hippocampus. (b–e) Segmentation results of our method with different initial seed points.

**Figure 9:**
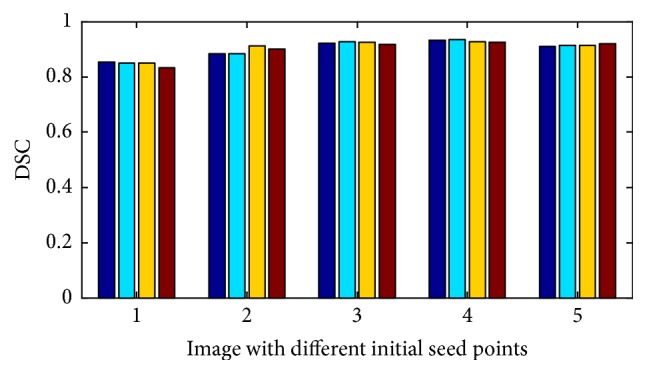
Segmentation accuracy of our method for images with different initial seed points.

In order to evaluate the robustness of our method, we apply it to the hippocampus images in Figure 5 with different initial seed points, as shown in Figure 8. In Figure 8, the original images and different initial seed points and the corresponding segmentation results are shown. In spite of huge difference of these seed points, the corresponding results are nearly the same, and the object boundaries are accurately obtained. To further illustrate the robustness to initialization of our method, the segmentation accuracy is quantitatively verified with DSC values in Figure 9. It can be seen that the *x*-axis represents five original images of hippocampus with four different initial seed points, and the *y*-axis represents the DSC value of each initialization. From the results we can see that the segmentation results obtained by each initial seed point are almost the same. Thus, experiment proved that the proposed model has a good robustness to the initial seed point.

## 4. Conclusion

In summary, we have introduced an approach to extract the pathway of hippocampus based on level set algorithm with an improved initialization using region growing, which allows us to optimize the location of hippocampus in a global sense. This model is derived from Li's model by incorporating the global Gaussian distributions with different means and variances into level set framework, avoiding the problems of boundary leakage low contrast and discontinuous boundaries of hippocampus images. Meanwhile, to solve the problem of initialization sensitivity, we have discussed and analyzed the adaptive region growing and morphological operations which are used to gain a rough segmentation result of hippocampus to initialize the level set function. Compared with Li's model, our method provides more accurate and efficient segmentation results that are closer to the manual segmentation results obtained by a specialist. In future work, we will further improve the segmentation efficiency by GPU acceleration and study the adaptive change of the shape constraint's effect according to the quality of the hippocampus images to acquire better segmentation results.

## Figures and Tables

**Algorithm 1:**
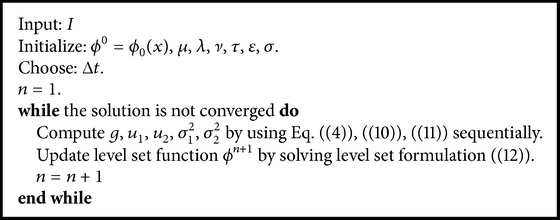
The implementation of the proposed level set algorithm.
